# *Helicobacter pylori* and colorectal neoplasia with emphasis on microbial mechanisms and causal evidence

**DOI:** 10.3389/fcimb.2026.1996461

**Published:** 2026-10-23

**Authors:** Jian Xu

**Affiliations:** Department of Gastroenterology, The People’s Hospital of Danyang, Affiliated Danyang Hospital of Nantong University, Zhenjiang, Jiangsu, China

**Keywords:** bacterial virulence, eradication therapy, gastric atrophy, gut microbiota, mucosal immunity, residual confounding

## Abstract

*Helicobacter pylori* (*H. pylori*) infection is associated with colorectal neoplasia in many observational studies, but a causal relationship and a preventive effect of eradication remain unestablished. This narrative review integrates clinical, microbial, experimental, and treatment evidence using four questions: which infection phenotype was measured, whether exposure preceded the colorectal outcome, whether a proposed intermediate was experimentally perturbed, and whether a defined intervention changed that outcome. Positive observational syntheses contrast with weaker or inconsistent prospective serological and genetic findings. Residual confounding, including socioeconomic and lifestyle differences, and non-equivalent exposure measurements limit causal interpretation. Gastric atrophy, endocrine changes, microbial remodeling, metabolites, and immune responses provide candidate links, but no complete infection-to-neoplasia sequence has been demonstrated in humans. Microbiota-dependent tumor promotion in genetically susceptible mice provides experimental support within that setting, whereas reduced tumor formation in an inflammation-driven model limits generalization. Neither model reproduces the duration and heterogeneity of human infection. Eradication findings depend on comparator selection, sufficient latency, and separation of randomized treatment assignment from successful clearance. A comparative appraisal links each proposed mechanism to its strongest available evidence, unresolved human causal link, and required validation. Infection status alone is not established as a basis for changing colorectal screening or claiming colorectal cancer prevention through eradication. Longitudinal human sampling, targeted tests of candidate intermediates, and long-term intervention analyses with explicit treatment comparisons are needed to establish clinical relevance.

## 1 Introduction

The established role of *Helicobacter pylori* (*H. pylori*) in gastric carcinogenesis has prompted sustained interest in its possible contribution to colorectal neoplasia. The clinical association, however, has been disputed from the outset. An early Italian series found higher seroprevalence particularly among patients with adenomas, whereas a matched United States (US) study found no association with adenoma or carcinoma (Meucci et al., 1997; Moss et al., 1995). A subsequent case–control investigation obtained different estimates when adenoma cases were compared with hospital controls rather than population controls, illustrating how recruitment can influence the apparent relationship (Breuer-Katschinski et al., 1999). Later meta-analyses have generally supported positive associations, but pooling does not remove the exposure misclassification, selection processes, or residual confounding of the original studies (Wu et al., 2013; Choi et al., 2020; Li et al., 2025). Colorectal neoplasia also encompasses different outcomes, from prevalent precursor lesions to incident colorectal cancer (CRC). Evidence concerning one outcome cannot automatically explain another, particularly when infection is measured only once or after the lesion has developed.

Proposed explanations have broadened from gastric physiology to interactions among infection, intestinal ecology, and host immunity. Earlier reviews emphasized gastrin, atrophic gastritis, direct bacterial detection, and epidemiological associations; more recent work integrates gut microbial changes with epithelial and immune responses (Coelho and Coelho, 2021; Engelsberger et al., 2024). This biological framework is attractive because a gastric exposure could influence colorectal tissue without persistent local colonization. Nevertheless, its individual components require different kinds of evidence. Tumor promotion in susceptible mice establishes an effect under defined experimental conditions, whereas human metabolomic associations identify candidate intermediates without demonstrating the complete pathway (Ralser et al., 2023; Chen et al., 2025). Active infection, persistent antibodies, virulence profiles, and gastric sequelae therefore represent related but non-equivalent exposures. Likewise, stool communities, mucosal responses, and circulating metabolites describe different biological compartments. Their joint consideration can clarify the hypothesis, provided that findings are not combined into a causal sequence that no study has directly tested.

Eradication offers an opportunity to examine whether infection-related effects are reversible, but treated and untreated patients may differ before therapy, and successful clearance is a post-treatment outcome rather than a randomized exposure (Shah et al., 2024; Han et al., 2026). A Bradford Hill-based review interpreted the relationship favorably, although whether eradication reduces CRC incidence or mortality remains unresolved (Frías-Ordoñez et al., 2025). Previous reviews already connected gastric physiology, microbiota, immunity, and eradication; the contribution here is a structured comparison of what each evidence type can establish, not a claim that this biological connection is newly proposed (Coelho and Coelho, 2021; Engelsberger et al., 2024). Four questions guide the synthesis: what infection phenotype was measured; whether it preceded the colorectal outcome; whether perturbing a proposed intermediate changed a relevant phenotype; and whether a defined intervention altered that outcome. Applying these questions exposes where evidence supports association or experimental pathway dependence without establishing human mediation or prevention. **Table 1** compares the strongest available mechanistic support, unresolved causal links, translational limits, and decisive validation tests; **Table 2** distinguishes treatment comparisons. This framework also separates etiological claims from possible applications in prediction, combined testing, and tumor targeting.

**Table 1:** Comparative appraisal of mechanistic evidence and validation priorities.

Proposed pathway	Strongest evidence and relative support	Unresolved link and clinical relevance	Essential validation	References
Atrophy and altered acidity	Human cross-sectional associations; prospective findings are inconsistent.	Acid change has not been shown to mediate subsequent colorectal lesions. Candidate phenotype, not a preventive target.	Serial infection, atrophy, acid, and microbiome measurements before incident lesions.	Chen et al., 2020, 2016
Gastrin and COX-2 signaling	Human hormone/tissue associations; prospective recurrent-adenoma evidence is negative.	Gastrin-dependent human carcinogenesis remains unproven; no basis for pathway-targeted CRC prevention.	Establish exposure timing; test pathway blockade and rescue at relevant hormone levels.	Georgopoulos et al., 2006; Hartwich et al., 2001; Robertson et al., 2009
Microbiota, mucus, and immunity	Germ-free and stool-transfer perturbations support microbiota-dependent tumor and epithelial phenotypes in *Apc*-mutant mice; some immune changes persist without microbiota.	Necessary organisms/functions and mediation in humans are unresolved. Experimental priority, not a clinical biomarker.	Selective microbial depletion/reconstitution with rescue; paired human microbial and mucosal sampling before lesions.	Ralser et al., 2023
Intestinal virome	Pre-tumor phage changes and inferred host/function links in mice; mainly associative.	A phage-dependent tumor-promoting function is unproven. No validated clinical application.	Manipulate candidate phages and measure bacterial function, host response, and tumor outcomes.	Luo et al., 2023
Metabolic intermediates	Prediagnostic human associations and statistical mediation; MR concerns methionine sulfone.	Temporal support exceeds cross-sectional profiles, but infection-driven mediation is unproven; no validated target.	Repeated sampling, mediation with confounder control, and metabolite perturbation/rescue.	Chen et al., 2025
Tumor immune context	Opposing tumor effects across *Apc* and AOM/DSS models; descriptive human immune correlates.	Direction depends on model; tumor initiation and treatment response are distinct. Human transferability is limited.	Harmonize strains, timing, and endpoints across models; test specific immune-cell dependence.	Ralser et al., 2023; Li et al., 2020; Wang et al., 2025
CagA and epithelial signaling	Cellular perturbation and microRNA rescue support a mechanism *in vitro*.	Physiological delivery to human colorectal cells and lesion initiation remain unproven.	Demonstrate relevant exposure; test dose-dependent effects in non-neoplastic tissue or organoids.	Zhong et al., 2021

Relative support describes experimental perturbation, temporal ordering, and directness within the reviewed studies; it is not a formal evidence-certainty grade. No pathway has a complete infection-to-neoplasia sequence established in humans. The validation column proposes future work and does not describe completed experiments. AOM/DSS, azoxymethane/dextran sodium sulfate; *Apc*, adenomatous polyposis coli; CagA, cytotoxin-associated gene A protein; COX-2, cyclooxygenase-2; CRC, colorectal cancer; MR, Mendelian randomization.

**Table 2:** Eradication and intervention evidence.

Exposure/intervention	Design	Outcome	Time window	Interpretive contribution	Principal bias	References
Clinical infection and treatment	Nationwide cohort	Incident CRC	Long term	Observational untreated vs treated infection; no randomized prevention estimate	Indication and adherence confounding	Shah et al., 2024
Eradication therapy	Swedish nationwide cohort	CRC risk	Long term	Post-treatment incidence association; baseline infection and treatment selection matter	Treatment selection; registry limits	Liu et al., 2024
Eradication therapy	Longitudinal follow-up	Incident CRC	Long term	Incidence vs general population; not an equivalent untreated infected comparator	Baseline risk and surveillance differ	Guo et al., 2023
Anti-*H. pylori* therapy	Meta-analysis/observational	CRC and adenoma	Mixed	Retrospective treatment contrasts; association rather than proof of prevention	Mixed designs; high heterogeneity	Li et al., 2025
Treatment × genetic susceptibility	*Post hoc* CRC analyses in two trial cohorts	CRC risk	Long term	Original-assignment contrasts assess treatment offer; clearance-based contrasts are selected	*Post hoc* CRC outcomes; subgroup multiplicity; clearance does not preserve randomization	Han et al., 2026
Infection/eradication	Critical review	Microbiome disruption/recovery	Short–long term	Separates acute perturbation from recovery	Heterogeneous, mostly non-cancer endpoints	Li et al., 2024

Treatment assignment, receipt, successful clearance, and general-population comparisons estimate different contrasts. Trial-cohort CRC analyses are *post hoc*, and clearance-based comparisons do not preserve randomization. No new treatment effect has been pooled for this review, and the listed findings do not establish definitive CRC prevention. CRC, colorectal cancer; *H. pylori*, *Helicobacter pylori*; vs, versus.

## 2 Literature search and selection

PubMed and Web of Science Core Collection were searched on 1 September 2026. The PubMed strategy combined Medical Subject Headings and title/abstract terms for *Helicobacter pylori* or Helicobacter infections with terms for colorectal, colonic, or rectal neoplasms, cancer, carcinoma, and colorectal adenoma. The Web of Science topic search required the infection and colorectal terms to occur within 50 words. The saved query strings contained no publication-year or language limits; additional interface filters were not documented. Exact queries and the documented selection workflow are provided in Supplementary Material S1. The searches yielded 794 PubMed records and 411 Web of Science records, with 874 records remaining after identifier/title-based deduplication.

Titles, abstracts, keywords, and publication types were relevance-ranked using a rule-based procedure, followed by assessment of accessible full texts for narrative synthesis. Selection prioritized studies linking infection or its treatment to colorectal adenoma, advanced neoplasia, or CRC, and studies testing relevant gastric, microbial, metabolic, epithelial, or immune intermediates. Human observational studies, trial-cohort analyses, genetic studies, and animal or cell experiments were considered; existing reviews supplied context and comparison. Reports lacking a relevant exposure, colorectal outcome, or explanatory pathway were not prioritized, and case reports received lower relevance scores. Detailed appraisal was restricted to accessible full texts. The final selection was purposive rather than exhaustive: this review does not claim dual independent screening, a registered systematic-review protocol, or formal risk-of-bias grading. Repeated populations, temporal ordering, exposure ascertainment, and the comparison actually estimated were assessed qualitatively, and no new effect estimates were pooled. Interpretation of treatment contrasts, time-related bias, and genetic causal inference was informed by methodological literature consulted during revision (Hernán and Hernández-Díaz, 2012; Hernán et al., 2016; Davies et al., 2018). This targeted consultation did not constitute a repeat database search.

## 3 Clinical and epidemiological evidence across colorectal outcomes

Clinical studies repeatedly associate *H. pylori* with colorectal neoplasia, but the magnitude and meaning of the association depend on the lesion examined and the way participants enter a study. **Table 3** summarizes selected designs and their principal interpretive limitations. In a meta-analysis of 55 studies, the pooled odds ratio (OR) was 1.47 (95% confidence interval (CI), 1.36–1.59) for adenoma, 1.77 (95% CI, 1.56–2.00) for advanced adenoma, and 1.59 (95% CI, 1.39–1.82) for CRC; the corresponding values of the heterogeneity statistic (I²) were 88%, 65%, and 89% (Li et al., 2025). **Figure 1A** presents these endpoint-specific estimates. These estimates neither track progression within the same individuals nor provide a uniform prediction across clinical populations, given the high heterogeneity. Other syntheses similarly report positive associations with precursor lesions or cancer, while differing in eligibility criteria, testing methods, and study composition (Kim et al., 2025; Chen et al., 2013; Zuo et al., 2020). **Figure 1B** compares published pooled estimates from 2013 and 2025 without conducting a new meta-analysis. Persistence of a positive pooled association over time is relevant, but overlapping primary studies prevent the syntheses from being counted as independent replications (Wu et al., 2013; Paiva Prudente et al., 2025). An umbrella review can expose disagreement among syntheses but cannot resolve their shared biases; interpretation should therefore prioritize study design, timing, and population independence over the number of positive reviews.

**Table 3:** Clinical and epidemiological evidence.

Study design	Population/outcome	Exposure assessment	Interpretive contribution	Principal limitation	References
Systematic review/meta-analysis	Adenoma and benign polyps	Heterogeneous tests	Overall association signal; heterogeneity calibration	Dependent on included-study quality	Kim et al., 2025
Umbrella review	Gastrointestinal cancers	Multiple exposure definitions	Evidence grading across overlapping syntheses	Inherited heterogeneity and overlap	Paiva Prudente et al., 2025
Systematic review/meta-analysis	East Asian CRC populations	Positive vs negative infection	Region-specific association synthesis	Limited geographic generalizability	Ma et al., 2023
Nationwide cohort	Incident CRC	Clinical infection record	Temporality and population-scale estimate	Residual confounding; exposure misclassification	Liu et al., 2019
Prospective European cohort	Incident CRC	Protein-specific antibodies	Virulence/antibody-profile stratification	Single serum measure; multiple testing	Butt et al., 2020
Retrospective cohort	Advanced colorectal neoplasia	*H. pylori*-specific IgG serology	Association with advanced lesions	Selection bias and retrospective design	Ryoo et al., 2020
Multi-cohort serologic study	Incident CRC	Protein antibody profile	Potential source of exposure heterogeneity	Population heterogeneity; multiple testing	Butt et al., 2019

Selected studies illustrate differences in outcome definition, exposure ascertainment, and design; the table is not an exhaustive list or a new evidence grading exercise. CRC, colorectal cancer; *H. pylori*, *Helicobacter pylori*; IgG, immunoglobulin G; vs, versus.

**Figure 1:**
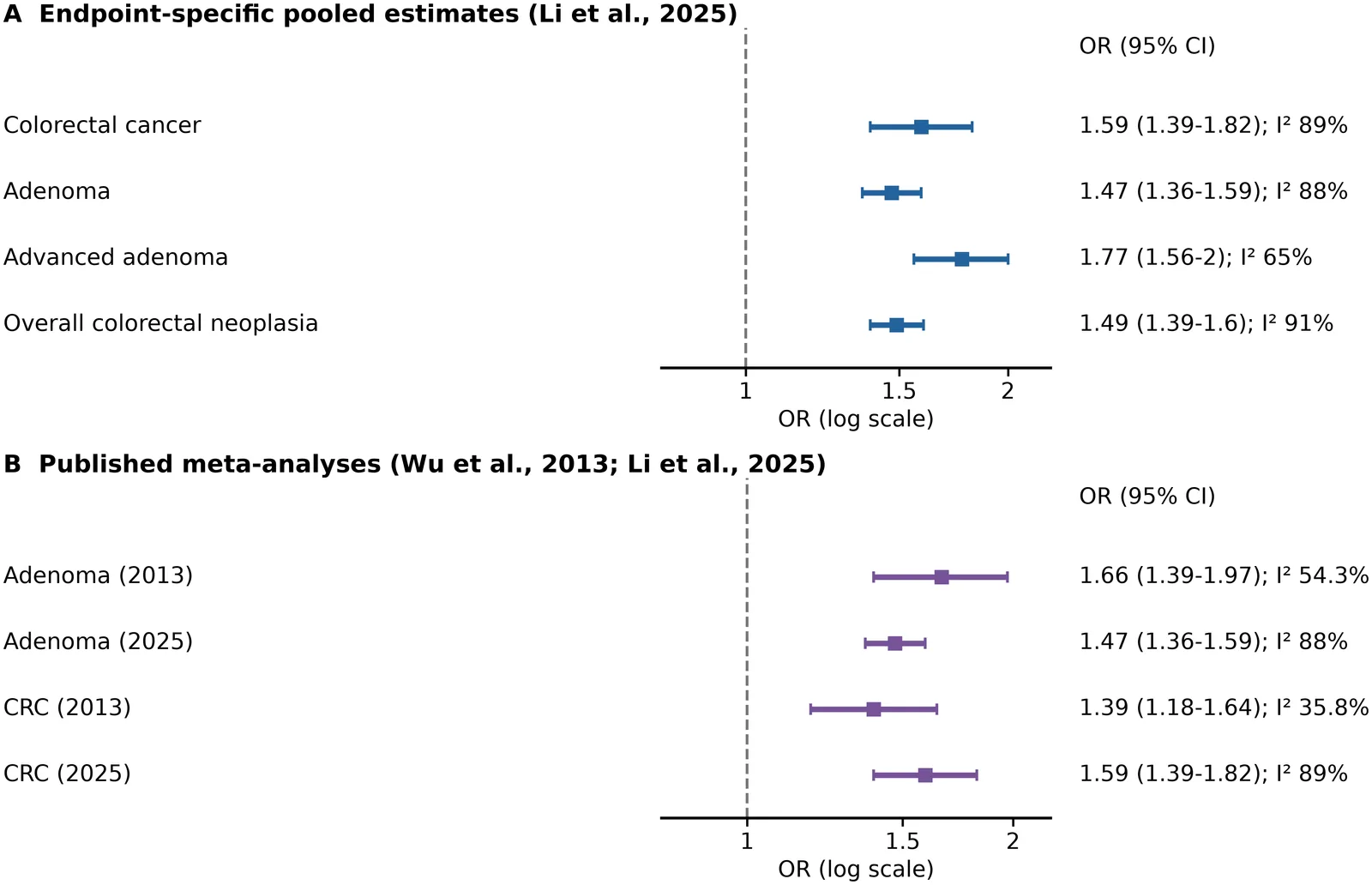
Quantitative evidence across the colorectal neoplasia continuum. **(A)** Pooled ORs for CRC, adenoma, advanced adenoma, and overall colorectal neoplasia reported by Li et al. (2025). **(B)** Published pooled estimates from Wu et al. (2013) and Li et al. (2025). Estimates and 95% CIs are displayed as reported and are not re-pooled. I² describes source-reported heterogeneity. Endpoints do not represent successive observations in the same individuals, and overlapping primary studies prevent treating the syntheses as independent replications. The reference line is OR = 1. CI, confidence interval; CRC, colorectal cancer; OR, odds ratio. I² is the percentage of variability in effect estimates attributable to between-study heterogeneity rather than sampling error.

At the primary-study level, a large US pathology database and a Korean screening study reported stronger associations for advanced or multiple adenomas, while a Chinese breath-test study also associated infection with adenomatous polyps (Sonnenberg and Genta, 2013; Nam et al., 2017; Chen et al., 2019). A more recent Chinese investigation distinguished advanced from low-risk adenomas, finding an association for the former but not the latter (Shan et al., 2026). These observations support outcome-specific analysis but do not establish accelerated malignant progression. Serrated lesions also warrant separate consideration: an association with serrated neoplasia in a retrospective study cannot be assumed to operate through the conventional adenoma pathway (Kumar et al., 2018). Conversely, a smaller endoscopy study found no significant association (Boyuk et al., 2019). Differences in lesion definitions and participant selection remain relevant when interpreting both positive and null findings.

Longitudinal studies add a temporal dimension but remain heterogeneous. A Taiwanese claims-based cohort reported an adjusted CRC hazard ratio (HR) of 1.87 (95% CI, 1.37–2.57), whereas a US Veterans cohort reported an HR of 1.18 (95% CI, 1.12–1.24) among individuals who underwent infection testing (Liu et al., 2019; Shah et al., 2024). Differences in populations and exposure records preclude interpreting these estimates as measurements of a universal effect. A large electronic-record analysis found an association with a recorded infection history, but its period-prevalence OR does not estimate the hazard of newly developing CRC (Boustany et al., 2023). In a separate retrospective cohort, infection was associated with metachronous advanced neoplasia over a median 41 months (adjusted HR, 1.74; 95% CI, 1.11–2.73) (Ryoo et al., 2020). Cross-study comparisons must account for baseline lesions, surveillance, and reasons for testing. Within this longitudinal evidence, prediagnostic serology provides a useful contrast. Overall associations were weaker in ten US cohorts and in a European cohort than might be expected from many retrospective syntheses, directing attention to antigen-specific responses and the meaning of a single antibody measurement (Butt et al., 2019, 2020). A positive African American endoscopy series and a null US Hispanic series likewise cannot establish ethnic effect modification through cross-study comparison alone, because recruitment, testing, and background risks differed (Brim et al., 2014; Patel et al., 2014). Regional findings in East Asian syntheses have similar limits (Ma et al., 2023). Prognosis must also remain separate from disease occurrence. An association with all-cause mortality in 110 selected CRC patients undergoing positron emission tomography/computed tomography does not establish an effect on cancer-specific survival or treatment response (Shmuely et al., 2018). Taken together, the clinical literature supports a recurring but population- and outcome-dependent signal. Its causal interpretation requires more precise characterization of infection and of the colorectal process being studied.

## 4 Infection phenotypes and direct colorectal detection

The term *H. pylori* positivity can refer to several biologically distinct states. Serology measures a host immune response that may persist after clearance, whereas urea breath and stool antigen tests more closely reflect active infection. Histology can add information about gastric pathology but is dependent on tissue sampling (Best et al., 2018). A single result cannot establish infection duration, its timing relative to a lesion, or sustained eradication; pooling different tests may obscure mechanistically important distinctions. In particular, an antibody-based association cannot be interpreted as evidence that viable organisms are currently acting in colorectal tissue. Protein-specific serology offers greater resolution, although the resulting signals have not been uniform. An early case–control study linked positivity for cytotoxin-associated gene A protein (CagA) among infected individuals to CRC (Shmuely et al., 2001). In the Southern Community Cohort Study, overall seropositivity was not associated with CRC, whereas selected antigen responses, including vacuolating cytotoxin A (VacA), were associated with risk (Epplein et al., 2013). An elderly, predominantly White prospective population again showed no overall serostatus association, with different antigen-specific findings and no dose–response pattern (Blase et al., 2016). These differences do not identify a validated high-risk strain or antibody profile: antigen responses mark exposure and immunity rather than the amount of bacterial product reaching the colon. Consortium analyses further illustrate this variability. **Figure 2A** shows estimates from ten US prospective cohorts, including the stronger VacA association among African American participants. **Figure 2B** shows European results, in which the fully adjusted VacA interval included the null (Butt et al., 2019, 2020). The panels retain study-specific estimates and are not a head-to-head comparison of assays or populations. A Spanish case–control study found no increased CRC risk associated with overall, CagA, or VacA seropositivity (Fernández de Larrea-Baz et al., 2017). A smaller investigation covering polyps, advanced adenomas, and CRC reported isotype- and antigen-dependent patterns, including an inverse association between immunoglobulin A responses to CagA and advanced adenoma (Genua et al., 2024). Interpretation must account for antibody class, antigen, endpoint, adjustment, and multiple testing rather than selecting only the strongest result from each study.

**Figure 2:**
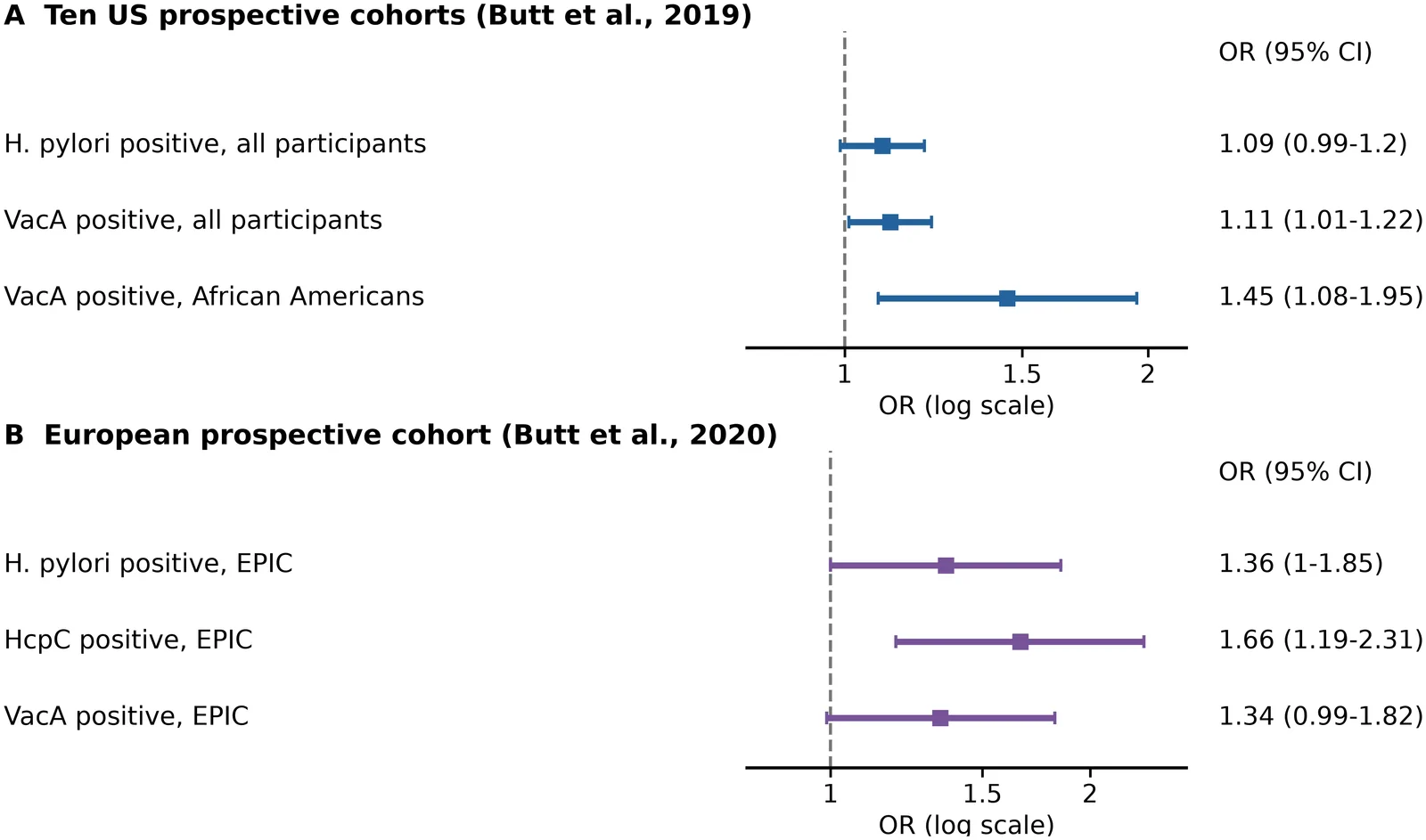
Exposure-specific associations with CRC. ORs and 95% CIs for overall *H. pylori* seropositivity and selected protein-specific responses. **(A)** Ten US prospective cohorts: conditional logistic regression accounting for matching variables, without additional covariate adjustment in the primary estimates (Butt et al., 2019). **(B)** European Prospective Investigation into Cancer and Nutrition: fully adjusted Model 2 estimates from **Table 1** (Butt et al., 2020). These responses include Helicobacter cysteine-rich protein C and VacA. Adjustment sets differ across panels, and estimates are not head-to-head comparisons of assays or populations. The reference line is OR = 1. CI, confidence interval; CRC, colorectal cancer; EPIC, European Prospective Investigation into Cancer and Nutrition; *H. pylori*, *Helicobacter pylori*; HcpC, Helicobacter cysteine-rich protein C; OR, odds ratio; US, United States; VacA, vacuolating cytotoxin A.

An informative infection phenotype should combine current infection status and prior treatment with strain-level virulence, host susceptibility, and measured microbial function. Antigen-specific serological studies measure immune responses, not recovered-strain genotypes or toxin delivery to colorectal cells (Butt et al., 2019; Genua et al., 2024). Trial-cohort analyses also considered host genetic susceptibility alongside antigen-defined infection profiles (Han et al., 2026). These measurements capture different biological dimensions. Future analyses should prespecify them and test interactions within a study rather than infer modification from comparisons between unrelated populations. Collapsing them into a single positive/negative exposure can obscure subgroup associations or create apparent inconsistency.

Direct detection in colorectal tissue addresses a different hypothesis: that bacterial material or viable organisms act locally. Histochemical and immunohistochemical studies have reported enrichment in adenoma tissue or more frequent staining in selected neoplastic groups (Zhuang et al., 2022; Jones et al., 2007; Mohamed et al., 2020). However, another immunohistochemical investigation described predominantly equivocal or granular staining and found no significant relationship with polyp size, location, or histological type (Soylu et al., 2008). A polymerase chain reaction study detected neither the *H. pylori* marker nor the *Streptococcus gallolyticus* (*S. gallolyticus*) marker in its 210-sample colorectal series (Mahmoudvand et al., 2017). These reports cannot be reconciled by vote counting: antigen staining, deoxyribonucleic acid (DNA) detection, and bacterial culture address different questions and differ in their sensitivity to processing, specificity, and contamination. Beyond reproducibility, local detection requires a temporal and functional explanation. Bacterial material could adhere preferentially to an altered mucosal niche after a lesion develops, rather than initiate the lesion. The small study combining serology and tissue measurements also found that correlations differed between normal and diseased mucosa, cautioning against using circulating antibodies as a surrogate for tissue abundance (Genua et al., 2024). A persuasive local mechanism would therefore require convergent spatial, molecular, and viability evidence, followed by evidence that the signal precedes neoplastic change and has a functional consequence. Direct and indirect hypotheses therefore remain open, making gastric physiology and the gut microbial environment important alternative routes to examine.

## 5 Gastric physiology and metabolic intermediates

Gastric pathology may identify a more informative exposure phenotype than infection status alone. In a retrospective cross-sectional study of 6, 018 health-check participants, infection accompanied by atrophic gastritis was associated with adenoma and advanced adenoma, whereas atrophy considered alone was not significant after adjustment (Chen et al., 2020). A Korean screening study similarly associated infection-related atrophy with advanced neoplasia, and a Japanese endoscopy study linked gastric atrophy with colorectal polyps (Lee et al., 2016; Kawahara et al., 2019). These observations are compatible with an indirect gastric pathway, but concurrent measurements of atrophy and colorectal lesions cannot establish causal mediation. Prospective and null findings provide an important counterpoint. The Epidemiological Study on Potentials for Prevention, Early Detection and Optimized Treatment of Chronic Diseases in the Elderly Population cohort found no association of infection or atrophy with incident colonic cancer over approximately ten years (Chen et al., 2016). A Japanese case–control study also found no overall CRC association with infection, gastrin, or atrophy, although it reported a rectal-cancer subgroup signal (Machida-Montani et al., 2007). Differences in lesion stage, exposure definition, recruitment, and residual confounding may contribute to this contrast with positive endoscopy studies. Reduced acid secretion and altered gastric ecology remain plausible intermediates, but the cited human studies primarily measured an atrophic phenotype rather than the complete sequence of infection, altered acidity, microbial change, and subsequent colorectal disease. **Table 1** distinguishes this proposed bridge from experimentally demonstrated pathway dependence.

The endocrine literature provides a related but incomplete line of evidence. Hypergastrinemia has been associated with distal adenomas, while findings on gastrin and cyclooxygenase-2 (COX-2) expression in CRC tissue suggest epithelial signaling hypotheses (Georgopoulos et al., 2006; Hartwich et al., 2001). Small case–control observations of circulating gastrin and ghrelin also varied with infection, tumor location, and stage (D'Onghia et al., 2007). Hormone measurements after cancer diagnosis may reflect disease-related physiology as well as prior exposure. In contrast, prospective analyses within adenoma chemoprevention trial populations found no increased recurrent-adenoma risk with higher gastrin (Robertson et al., 2009). The serology comparison in that setting was not a randomized eradication experiment. Thus, the available associations and proliferative signals do not establish gastrin-mediated colorectal carcinogenesis.

Metabolic comorbidity introduces further possibilities and shared determinants. Infection combined with metabolic syndrome or elevated glycated hemoglobin was associated with greater adenoma prevalence in cross-sectional studies (Lin et al., 2010; Hu et al., 2017). These designs cannot establish which exposure occurred first or determine whether the metabolic condition is a mediator, a confounder, or a correlated consequence. A newer study reported statistical mediation through metabolic dysfunction-associated steatotic liver disease, but its cross-sectional design and nonsignificant interaction test limit causal interpretation (Cai et al., 2026). Contemporaneous mediation estimates cannot show that modifying the proposed intermediate changes colorectal risk, particularly when mediator and outcome share incompletely measured determinants. By contrast, prediagnostic metabolomics adds temporal information. In one study, infection-associated metabolites statistically mediated part of the association with CRC risk in males, although the estimates had wide CIs; its accompanying genetic analysis concerned methionine sulfone rather than infection itself (Chen et al., 2025). This work links exposure-related metabolic variation to subsequent disease more directly than a cross-sectional comparison, while still leaving the origin and causal role of the metabolite unresolved. The genetic result cannot independently validate every earlier step of the proposed infection pathway. Overall, gastric pathology, endocrine markers, and circulating metabolites identify candidates for longitudinal and interventional study. These candidates can guide measurement but are not validated targets for CRC prevention. No cited human study establishes all of the following in sequence: infection, an infection-induced change in gastric physiology or microbial function, a consequent epithelial or immune alteration, and later colorectal neoplasia. Prediagnostic metabolomics provides stronger temporal evidence than concurrent tissue or stool comparisons, but neither statistical mediation nor an instrument for methionine sulfone proves that infection generated the relevant metabolite (Chen et al., 2025). Relative support is therefore pathway-specific. Microbiota-dependent tumor and epithelial phenotypes have experimental perturbation support in susceptible mice, although not all immune changes depend on the microbiota (Ralser et al., 2023). Metabolic intermediates have prospective human associations (Chen et al., 2025), whereas endocrine and atrophy-related links are supported mainly by observational and tissue findings (Hartwich et al., 2001; Georgopoulos et al., 2006; Chen et al., 2020). Their translational relevance depends on completing the missing human links identified in **Table 1**.

## 6 Gut microbial changes and pathway dependence

The gut microbiome offers a plausible connection between a gastric infection and colorectal tissue responses. Its relevance depends, however, on whether observed ecological differences precede disease and contribute to a functional outcome. In a 60-participant stool-profiling study, infected individuals differed in bacterial and fungal composition, but participants were not followed to determine subsequent CRC development (Dash et al., 2019). A newer cross-sectional study compared CRC patients with healthy groups defined by infection status and also identified community differences (Qi et al., 2026). These profiles establish neither directionality nor microbial mediation of the infection–cancer association. An intermediate community profile need not represent a transitional disease stage: groups may differ in other characteristics, and cancer itself may alter the microbiota. Repeated sampling before a colorectal endpoint would be needed to distinguish these explanations. Spatial sampling adds another layer of uncertainty. Stool, gastric biopsies, normal colorectal mucosa, and tumor tissue represent different habitats, and a fecal taxonomic change need not imply an equivalent change in mucosal function. Reviews integrating intestinal microbiota and immunity have already emphasized these connected compartments (Engelsberger et al., 2024). The unresolved task is to connect specific organisms or functions to host responses within the same setting.

Animal experiments provide a means of testing dependence on the microbial environment. In infected mice carrying a mutation in the adenomatous polyposis coli (*Apc*) gene, the tumor-promoting phenotype was attenuated under germ-free conditions; stool-transfer experiments additionally supported a contribution of infection-associated microbiota (Ralser et al., 2023). Among the pathways reviewed, these perturbations provide comparatively direct support for microbiota-dependent tumor and epithelial phenotypes within a susceptible host. However, increased T-cell infiltration and reduced regulatory T-cell abundance persisted under germ-free conditions, indicating that not all immune changes were microbiota-dependent (Ralser et al., 2023). They do not identify a single necessary organism or demonstrate that the same mediation occurs in humans. Experiments using CagA-positive strains in another mouse setting produced colorectal inflammatory lesions and altered bacterial communities, but these endpoints are not established CRC incidence or human cohort evidence (Cui et al., 2025). Within this microbial environment, the intestinal virome may also contribute. Profiling in infected mice identified temperate-phage expansion and bacterial–viral associations before tumor onset (Luo et al., 2023). The pre-tumor timing is informative, yet predicted bacterial hosts and inferred gene functions remain distinct from experimental evidence that a phage causes a tumor-promoting community change. Selective depletion and reconstitution of candidate organisms or phages, with rescue of the host phenotype, would test functional dependence. **Table 1** distinguishes this proposed validation from experiments already performed and from human evidence still needed.

Human multi-pathogen observations also require careful interpretation. Joint seropositivity to *H. pylori* VacA and *the S. gallolyticus* protein antigen Gallo2178 was associated with CRC in a US cohort consortium, but the interaction test was borderline and the analysis reused a population represented elsewhere in the serological literature (Epplein et al., 2020). Joint antibody responses do not demonstrate physical cooperation between the organisms in colorectal tissue, and this analysis cannot be counted as independent replication of the consortium’s earlier findings. Combined microbial exposures warrant spatial and functional testing, but bacterial, viral, and metabolic findings cannot substitute for one another within a proposed pathway. Treatment-related ecology requires a further distinction from infection-associated changes. Eradication exposes the gastrointestinal ecosystem to antibiotics and acid suppression as well as removing the pathogen, and changes differ across compartments and follow-up intervals (Li et al., 2024). A community that changes after treatment does not by itself indicate either recovery of a cancer-protective state or emergence of a harmful one. The relevant test would link the ecological change to later colorectal outcomes while accounting for the treatment received and the persistence or clearance of infection. Together, the human and experimental evidence supports the microbiome as a candidate intermediary, with the strongest mechanistic inference currently restricted to particular experimental settings. Repeated human measurements and targeted perturbation of proposed functions are needed to determine which of these findings belong to a reproducible colorectal pathway.

## 7 Epithelial and immune responses in experimental context

Epithelial and immune responses provide a possible functional link between microbial changes and tumor behavior. In infected *Apc*-mutant mice, tumor promotion was accompanied by goblet-cell loss, signal transducer and activator of transcription 3 activation, and regulatory T-cell changes, with related epithelial and immune findings in human biopsies (Ralser et al., 2023). The biopsy observations support biological plausibility but do not reproduce the intervention or establish temporal sequence in patients. CagA-positive strain experiments in a different mouse setting likewise supported inflammatory and microbial perturbations without establishing a neoplastic endpoint (Cui et al., 2025). Importantly, infection reduced tumor formation and tumor-associated macrophage infiltration in an azoxymethane/dextran sodium sulfate (AOM/DSS) model of colitis-associated cancer (Li et al., 2020). Genetic susceptibility and chemically induced epithelial injury therefore produce different contexts in which infection-associated immune changes may have opposing effects. These studies are not interchangeable replications of a single mechanism. Neither their accelerated tumor development nor their experimentally controlled infections reproduce decades-long human exposure, changing diet, treatment histories, and microbial communities. Direct comparisons using harmonized bacterial strains, host backgrounds, infection timing, and tumor endpoints are required before the source of the disagreement can be identified. Human associations between infection and macrophage expression in neoplastic tissue remain descriptive (Wang et al., 2025). Model dependence is also evident outside *H. pylori* research: reducing oxidative DNA damage limited tumors in inflammatory CRC models but not in a mismatch-repair-deficient model (Irrazabal et al., 2020). That study supplies a mechanistic analogy, not infection-specific proof.

Cell experiments can interrogate a more specific molecular step. CagA-positive bacterial coculture and CagA overexpression enhanced proliferation, invasion, and autophagy in colon cancer cells in association with reduced microRNA-125b-5p; restoring the microRNA counteracted these effects *in vitro* (Zhong et al., 2021). Such perturbation supports a cellular mechanism within the tested system. Its interpretation for human disease still depends on whether a comparable bacterial product reaches the relevant cells at a biologically meaningful exposure. An experiment using overexpression does not establish that this exposure occurs during gastric infection, nor does a response in an established cancer cell line demonstrate initiation of a colorectal lesion. These qualifications define the next translational tests.

Human tissue and computational studies identify additional candidates but answer less direct causal questions. Studies of phosphorylated ribosomal protein S6 kinase 1 and of the expression of genes encoding tissue inhibitor of metalloproteinases 3, elastin, the Kirsten rat sarcoma viral oncogene homolog, and the B-Raf serine/threonine kinase report associations rather than evidence that infection induces driver mutations; the latter study measured transcripts by reverse transcription quantitative polymerase chain reaction, not mutation status (Jia and Wan, 2023; Zeadan et al., 2026). Shared-gene analyses across public infection and CRC datasets similarly prioritize inflammatory pathways without demonstrating an infection-to-tumor sequence within individuals (Wei et al., 2026). Shared inflammatory expression signatures can guide experiments but do not independently confirm a cross-organ mechanism. For immune findings after cancer develops, the relevant outcome must also be distinguished. In human CRC, inducible T-cell costimulator-positive regulatory T cells and infection serology have been associated with immune phenotypes and selected prognostic outcomes. The preoperative eradication observations in that study concerned gastric cancer patients, not a CRC treatment trial; a survival benefit from eradication in CRC therefore remains unproven (Urakawa et al., 2021). Infected mice bearing tumors derived from the murine colon adenocarcinoma cell line MC38 or melanoma also showed impaired immunotherapy responses, whereas the accompanying clinical cohorts involved non-small-cell lung cancer rather than CRC (Oster et al., 2022). Tumor initiation, progression, prognosis, and response to immunotherapy are therefore distinct outcomes, even when immune processes appear in each. Collectively, these studies support context-dependent epithelial and immune effects. Whether removing infection or perturbing an intermediate improves a clinically relevant colorectal outcome must be established through the corresponding intervention comparison.

## 8 Eradication and the interpretation of treatment evidence

Eradication studies offer an opportunity to test reversibility, but the causal question depends on the comparison. **Table 2** distinguishes randomized treatment assignment, treatment receipt, successful clearance, and incidence relative to a general population; **Figure 3** retains the original effect measures and reference groups. For precursor lesions, a retrospective cohort with repeated endoscopy reported more adenoma development under persistent infection than after successful eradication (Hu et al., 2019). Clearance is determined after treatment and may depend on adherence, antibiotic resistance, host characteristics, and subsequent testing. Comparing successful clearance with persistent infection therefore does not preserve randomized comparability, even when participants originated in a trial (Hernán and Hernández-Díaz, 2012). The US Veterans cohort extends the observational evidence to CRC incidence and mortality. Untreated versus treated infection was associated with higher hazards of incident and fatal CRC, as displayed in **Figure 3A** (Shah et al., 2024). Treatment was not randomized, and differences in treatment decisions, healthcare use, and surveillance preclude interpreting these estimates as definitive preventive effects. In observational treatment analyses, time zero and exposure classification must also be aligned: assigning future treatment or clearance status at baseline can introduce immortal-time or other time-related selection bias (Hernán et al., 2016). This is a design consideration, not a claim that every study cited here made this error.

**Figure 3:**
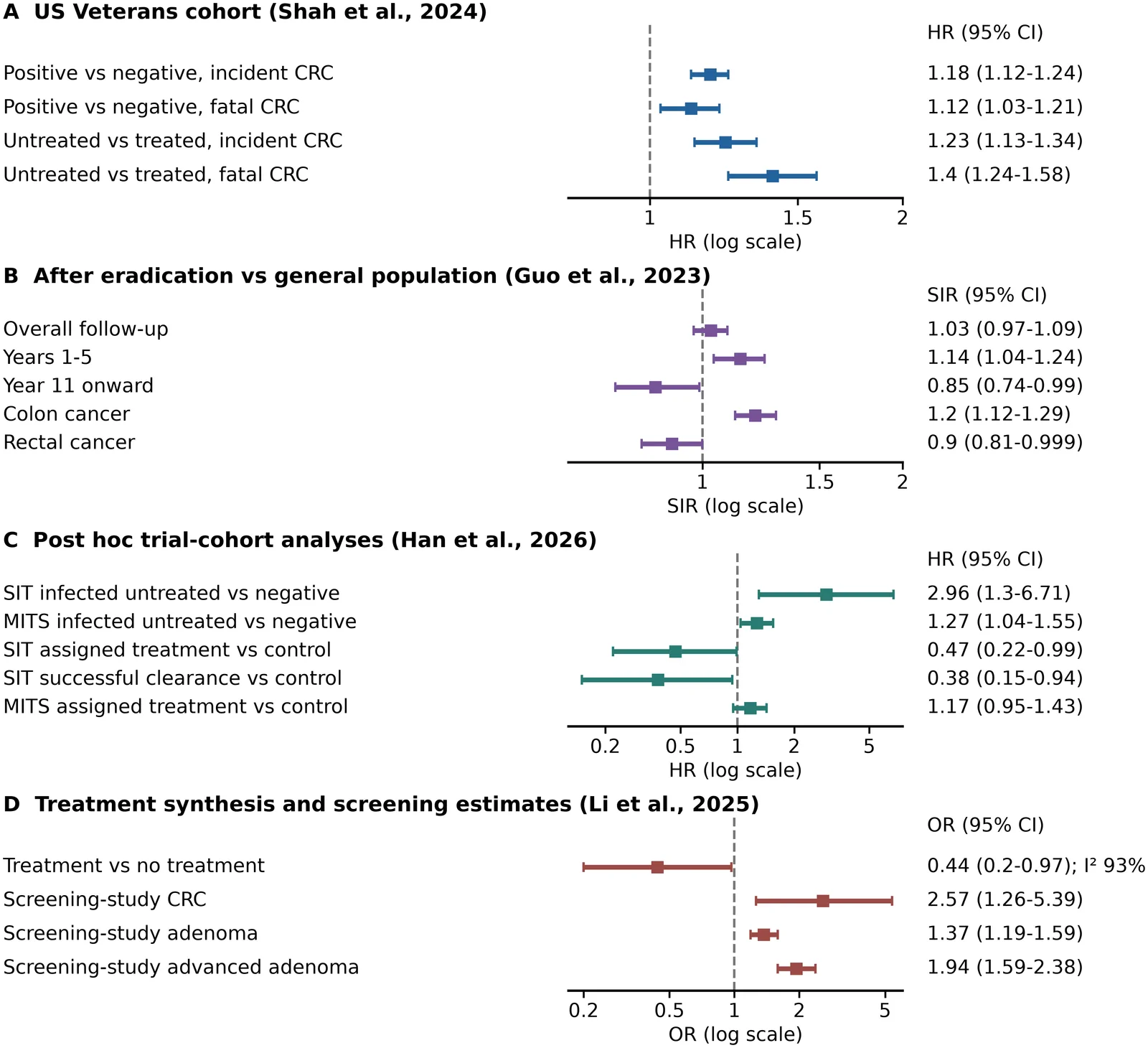
Infection, treatment, and colorectal outcomes across study designs. **(A)** Adjusted HRs for incident and fatal CRC, comparing positive with negative infection tests and untreated with treated infection in a US Veterans cohort (Shah et al., 2024). **(B)** SIRs after eradication relative to the Hong Kong general population, taken from **Table 1** of Guo et al. (2023); the first-five-year estimate follows the table and Results section rather than the inconsistent abstract. **(C)** HRs in the SIT and the MITS (Han et al., 2026). Assigned-treatment contrasts compare the original randomized groups; infection contrasts are observational, and successful clearance is a selected post-treatment comparison. None of these panels establishes a uniform CRC-preventive effect. CRC analyses are *post hoc*. **(D)** Treatment versus no-treatment OR from three retrospective studies and separate cross-sectional infection associations in the screening study reported by Li et al. (2025). Treatment and cross-sectional infection estimates address different questions. All intervals are 95% CIs. Metrics, reference groups, and time windows remain within their respective panels; estimates are not pooled across designs. The reference value is 1 for each ratio measure. CI, confidence interval; CRC, colorectal cancer; HR, hazard ratio; MITS, Mass Intervention Trial in Linqu; OR, odds ratio; SIR, standardized incidence ratio; SIT, Shandong Intervention Trial; US, United States; vs, versus. I² is the percentage of variability in effect estimates attributable to between-study heterogeneity rather than sampling error.

Population-standardized comparisons address another question. In Hong Kong, the overall standardized incidence ratio (SIR) after eradication was 1.03 (95% CI, 0.97–1.09) relative to the general population, with an early excess and lower incidence after 11 years (**Figure 3B**) (Guo et al., 2023). A Swedish registry study did not find a clear, consistent reduction across follow-up intervals (Liu et al., 2024). More recently, a Korean cohort of 931, 585 treated individuals reported lower CRC incidence and mortality than the general population over a mean 12.4 years, with an SIR of 0.66 and a standardized mortality ratio of 0.51 (Jung et al., 2026). Population-standardized comparisons do not recreate untreated groups with equivalent infection histories and baseline risks. Healthcare use and the exclusion of cancers diagnosed within the first year in the Korean study are among the features that need to accompany interpretation of the apparent benefit. Treatment syntheses likewise inherit the limitations of their component studies. The 2025 meta-analysis reported a treatment OR of 0.44 (95% CI, 0.20–0.97; I² = 93%) from three retrospective studies (Li et al., 2025). **Figure 3D** separates that contrast from the paper’s cross-sectional infection associations. The wide interval, small number of treatment studies, and high heterogeneity warrant caution about a uniform preventive effect. Pooling retrospective treatment contrasts does not make treatment randomized, and the magnitude of a pooled OR cannot be directly compared with an SIR or a time-to-event HR as if they estimated the same quantity. The apparent benefit warrants better intervention evidence.

Analyses within randomized trial cohorts provide a different basis for comparison. In the Shandong Intervention Trial (SIT), assigned treatment was associated with lower CRC risk over 29.4 years (HR, 0.47; 95% CI, 0.22–0.99). In the Mass Intervention Trial in Linqu (MITS), there was no overall reduction after 13.8 years (HR, 1.17; 95% CI, 0.95–1.43) (**Figure 3C**) (Han et al., 2026). These are *post hoc* CRC analyses within established trial cohorts. Comparisons by original assignment estimate the effect of offering the treatment regimen under the trial conditions; they do not isolate the effect of bacterial clearance (Hernán and Hernández-Díaz, 2012). Infection-positive versus infection-negative contrasts are observational, and conditioning on successful eradication may select participants according to adherence, resistance, and follow-up testing. Genetic and antigen-defined subgroups require interaction tests and attention to multiplicity rather than inference from significance within one stratum. Short follow-up may miss effects on slowly developing lesions, whereas early diagnoses may reflect lesions already present at treatment or differential surveillance. However, the difference between the two trials cannot be attributed to follow-up duration alone, because populations and other design features also differ. Prespecified lag analyses, baseline lesion information, and comparable surveillance would improve interpretation. Antibiotics and acid suppression may themselves alter microbial ecology, so a treatment effect cannot automatically be assigned to removal of *H. pylori* (Li et al., 2024; Engelsberger et al., 2024). Future studies should prioritize long-term analyses by randomized assignment, with clearance-based analyses reported separately. Current findings do not establish a reproducible preventive effect across populations or colorectal endpoints.

## 9 Causal inference and sources of heterogeneity

The causal argument draws support from several kinds of evidence, but their contributions are unequal. Positive observational syntheses establish a recurring association, while infection experiments establish tumor-promoting potential under specified conditions (Li et al., 2025; Ralser et al., 2023). Neither result alone demonstrates a universal human effect. A Bradford Hill-based review interpreted the accumulated findings favorably while calling for confirmation of whether eradication reduces CRC outcomes (Frías-Ordoñez et al., 2025). This appraisal interprets existing studies rather than estimating intervention benefit, and must account for shared populations and differing exposure definitions when judging consistency, temporality, and plausibility. Genetic analyses require the same alignment between the proposed exposure and its measurement. A bidirectional analysis found no clear association of genetically predicted overall seropositivity, CagA antibodies, or VacA antibodies with CRC (Luo et al., 2023). A subsequent two-sample analysis using anti-*H. pylori* immunoglobulin G (IgG) levels also produced null estimates, but antibody instruments do not directly represent active infection, its duration, or treatment response (Wang et al., 2025). Another study combined a prospective serological analysis that was not robustly associated with CRC with Mendelian randomization (MR) of peptic ulcer disease rather than infection (Wan et al., 2025). Conversely, the metabolomic study’s genetic analysis concerned methionine sulfone (Chen et al., 2025). These analyses test different exposures: null antibody estimates do not exclude every infection effect, while a metabolite estimate cannot establish the entire upstream pathway.

Residual confounding remains a competing explanation for at least part of the association. Socioeconomic circumstances and early-life living conditions are related to infection acquisition, while diet, smoking, adiposity, medication exposure, and access to screening can also influence colorectal risk or detection (Engelsberger et al., 2024; Butt et al., 2019; Shah et al., 2024). Adjustment for age and sex alone cannot account for these differences, and inconsistent measurement across studies limits what multivariable adjustment achieves. These factors may influence associations in either direction rather than necessarily explain them away. Prediagnostic serology improves temporal ordering but measures an immune response rather than active infection (Butt et al., 2019, 2020). Genetic analyses can reduce some conventional confounding only when the instruments are associated with the exposure, are independent of exposure–outcome confounders, and affect the outcome only through that exposure (Davies et al., 2018). The infection-related genetic studies examined antibody phenotypes, which do not directly represent every dimension of active or past infection (Luo et al., 2023; Wang et al., 2025). Discordance between these designs and retrospective syntheses therefore reduces confidence in a broad causal claim without proving that every infection phenotype has no effect. Better-controlled longitudinal studies should measure socioeconomic and lifestyle determinants, treatment history, and surveillance alongside repeated infection assessments.

Effect modification requires equally specific interpretation. In Korean health-check data, the infection association with advanced neoplasia was concentrated among younger smokers and accompanied by a reported three-way interaction (Park et al., 2018). A newer multicenter study described different sex-stratified patterns for seropositivity and intestinal metaplasia, but its formal interaction tests were nonsignificant (Kim et al., 2026). Significance in one stratum and not another is therefore insufficient to establish a sex-specific biological effect. Similar caution applies to metabolic syndrome, glycemia, and steatotic liver disease: their co-occurrence with infection and adenoma may reflect shared causes, correlated exposures, or candidate intermediates (Lin et al., 2010; Hu et al., 2017; Cai et al., 2026). Interpretation must follow study design and temporal information. Beyond effect modification, heterogeneity reflects the quantity being estimated. Prevalent-lesion odds, subsequent CRC hazards, and incidence relative to a general population are not alternative scales for the same clinical contrast. **Figure 1** and **3** make these differences visible without combining them into a new pooled result. Selection into infection testing or endoscopy and variation in surveillance remain relevant even in large datasets (Liu et al., 2019; Shah et al., 2024; Boustany et al., 2023). Evidence integration is most defensible when population, exposure, outcome, and comparator are aligned before results are compared. On that basis, association and experimental plausibility are supported, but the magnitude, pathway specificity, and reversibility of a human causal effect remain insufficiently established for broad colorectal recommendations.

## 10 Clinical translation and validation requirements

One potential application is to use infection-related information for colorectal risk assessment. Published models have incorporated serology into advanced-neoplasia scores for younger asymptomatic adults and infection status into broader adenoma nomograms (Park et al., 2017; Li et al., 2022). A newer model integrating gastric-related indicators reported an internal-validation area under the curve of 0.837, with lower performance for several established scores in the same dataset (Chen et al., 2026). These results warrant further evaluation: an internal split is not external validation, and predicting contemporaneous lesions differs from estimating future cancer risk. Clinical value depends on the intended decision; complete-model comparisons do not isolate the contribution of infection, and improved discrimination does not establish patient benefit. Further evaluation should examine calibration, transportability, decision thresholds, and the consequences of allocating colonoscopy differently. Protein-specific responses could be candidate predictors, but inconsistent antigen and isotype findings argue against treating any current panel as a deployable CRC test (Epplein et al., 2013; Genua et al., 2024). Validation should therefore separate the predictive usefulness of a measured marker from claims about the biological mechanism that generated it.

On the evidence synthesized here, infection status alone does not provide sufficient justification for initiating earlier colonoscopy, shortening surveillance intervals, or recommending eradication specifically to prevent CRC. This limitation concerns the colorectal inference drawn here; it does not argue against treatment for established *H. pylori*-related indications. A marker could eventually improve prediction without itself being causal, but it would still need incremental predictive value and demonstrated clinical utility. Conversely, biological plausibility or successful bacterial clearance is not sufficient evidence of colorectal prevention. These distinctions keep present clinical decisions separate from the research priorities summarized in **Table 1**.

Combined testing provides a distinct service-delivery opportunity. A prospective diagnostic study measured *H. pylori* stool antigen using material collected in fecal immunochemical test (FIT) tubes and found promising diagnostic performance and convenience (Nieuwenburg et al., 2022). This involved an additional antigen assay, not detection of infection by the fecal-hemoglobin assay itself. A Taiwanese economic analysis subsequently modeled adding one-time stool antigen testing to biennial FIT using evidence from a pragmatic trial; projected outcomes depended on adherence, costs, prevalence, and both gastric and colorectal disease (Lee et al., 2026). Combined-program value is distinct from etiological evidence and does not independently establish CRC prevention by eradication. Other applications use bacterial components without treating infection as a risk factor. Fluorescent recombinant Helicobacter outer membrane protein Q labeled carcinoembryonic antigen-related cell adhesion molecule-expressing colorectal tumors and metastases in orthotopic mouse models, demonstrating targeting potential (Hollandsworth et al., 2020). This does not establish a clinical imaging benefit or show that infection caused the tumors. Similarly, improved microbial recovery or treatment tolerance after an adjunct cannot be equated with reduced colorectal neoplasia (Li et al., 2024). Detection, risk stratification, combined testing, and tumor targeting require distinct comparators and outcome validation; success in one application does not validate the others.

## 11 Discussion and research priorities

The four-question framework separates exposure definition, temporal ordering, experimental pathway dependence, and intervention reversibility. Previous syntheses already integrated gastric physiology, microbiota, immunity, and eradication (Coelho and Coelho, 2021; Engelsberger et al., 2024). The additional insight is that agreement at one level cannot substitute for evidence at another: a positive pooled association does not establish microbial mediation, a mouse perturbation does not establish a human effect, and successful clearance does not define a randomized treatment contrast. **Table 1** makes the relative support explicit. Germ-free and stool-transfer experiments provide comparatively direct support for microbiota-dependent tumor and epithelial phenotypes in susceptible mice, but some immune changes persist without the microbiota and human mediation remains unresolved (Ralser et al., 2023). Prediagnostic metabolomic findings improve temporal ordering without establishing the origin or causal role of every metabolite (Chen et al., 2025). Atrophy and endocrine findings remain inconsistent across human designs (Chen et al., 2016, 2020; Georgopoulos et al., 2006; Robertson et al., 2009), while the phage evidence is primarily associative (Luo et al., 2023). This comparison is a qualitative appraisal of study design and directness, not a formal certainty score or a validated prediction framework. Opposing animal results and divergent treatment comparisons define the limits of the current causal argument rather than being exceptions to a proven pathway (Li et al., 2020; Ralser et al., 2023; Han et al., 2026).

Future human studies should measure infection, candidate intermediates, and subsequent colorectal outcomes within the same individuals. Cross-sectional microbial and metabolic studies cannot establish the full temporal sequence (Qi et al., 2026; Cai et al., 2026). We therefore propose repeated active-infection and antibody assessments, gastric pathology, and microbial or metabolomic sampling before adjudicated lesions to distinguish persistent exposure, gastric sequelae, and consequences of existing disease. Serial mediation analyses should account for socioeconomic circumstances, diet, medication use, and time-varying determinants of both the intermediate and the outcome. Candidate microbial functions and metabolites then require perturbation and rescue experiments at biologically relevant exposures, with comparison across susceptible and inflammatory models. Treatment analyses should preserve randomized assignment where available, distinguish clearance from allocation, and align time zero and surveillance (Hernán and Hernández-Díaz, 2012; Hernán et al., 2016). The internally validated prediction model provides a candidate for further assessment, not evidence of transportability or improved clinical outcomes (Chen et al., 2026). We propose geographical and temporal external validation, explicit comparison with and without infection variables, and prospective assessment of whether changed decisions improve outcomes. These tests address different uncertainties and should not be treated as interchangeable confirmations.

The review itself also limits inference. Selection was purposive and full-text appraisal depended on availability; the database strategies and relevance-ranking approach may have missed pertinent studies. The archived Web of Science proximity restriction could miss records in which the infection and colorectal terms occur far apart. The initial database searches were not an exhaustive search update to the revision date. No dual independent screening or formal outcome-specific certainty assessment is claimed. Overlap among primary studies in meta-analyses and reuse of the same US cohort consortium across publications limit independent replication (Butt et al., 2019; Epplein et al., 2020; Paiva Prudente et al., 2025). The framework organizes these limitations and identifies discriminating tests; it cannot resolve human causality from the existing literature alone.

## 12 Conclusion

*H. pylori* infection is associated with colorectal neoplasia in many observational studies, but residual confounding and inconsistent prospective and genetic findings prevent a firm conclusion about human causality. Experimental evidence supports microbiota-dependent and immune-mediated effects in particular settings, with opposing tumor outcomes across animal models and no complete mechanistic sequence established in humans. Treatment assignment, treatment receipt, successful clearance, and general-population comparisons answer different questions; existing eradication findings do not establish a uniform CRC-preventive effect. Infection status alone should therefore not be used, on the basis of this review, to change colorectal screening or surveillance. The immediate contribution is a comparative framework linking each proposed pathway to the evidence it supports, the causal step still missing, and the validation needed before clinical translation.
